# High-Performance Two-Stage DC/DC Converter Based on LADRC-PI Hybrid Control for PEM Electrolyzer Hydrogen Production

**DOI:** 10.3390/mi16060665

**Published:** 2025-05-31

**Authors:** Qingshuai Yu, Zhenao Sun, Yetong Han, Tuanlong Zhang, Rongxing Zhang, Muhua Lin

**Affiliations:** College of Information Science and Engineering, Northeastern University, Shenyang 110819, China; yuqingshuai6510@163.com (Q.Y.); 2310255@stu.neu.edu.cn (Y.H.); 2371037@stu.neu.edu.cn (T.Z.); 2300796@stu.neu.edu.cn (R.Z.); 18320339701@163.com (M.L.)

**Keywords:** hydrogen conversion converter, proton exchange membrane (PEM) electrolyzer, LADRC, ripple suppression

## Abstract

While DC/DC converters for water electrolysis systems have been widely investigated, they inherently face a critical compromise between wide voltage regulation capabilities and dynamic response characteristics. This study is based on a two-stage hybrid topology (TSIB-TPLLC) that synergistically combines a two-phase interleaved buck converter with a three-phase LLC resonant converter to resolve this challenge. The first-stage interleaved buck converter enables wide-range voltage regulation while reducing input current ripple and minimizing intermediate bus capacitance through phase-interleaved operation. The subsequent three-phase LLC stage operates at a fixed resonant frequency, achieving inherent output current ripple suppression through multi-phase cancellation while maintaining high conversion efficiency. A dual-loop control architecture incorporating linear active disturbance rejection control (LADRC) with PI compensation is developed to improve transient response compared to conventional PI-based methods. Finally, a 1.2 kW experimental prototype with an input voltage of 250 V and an output voltage of 24 V demonstrates the converter’s operational feasibility and enhanced steady-state/transient performance, confirming its suitability for hydrogen production applications.

## 1. Introduction

Amid the global shift toward decarbonized energy systems, hydrogen has emerged as a critical secondary energy carrier, linking renewable energy generation with end-use applications. Its scalability positions it as a strategic focus in international energy technology competition [1,2,3]. Proton exchange membrane (PEM) water electrolysis has emerged as an ideal solution for converting intermittent renewable energy derived from wind and solar power sources owing to its rapid dynamic response characteristics and high-purity hydrogen production capabilities. As the core power conversion interface in PEM electrolyzers, the DC/DC converter must achieve high efficiency, strong fault tolerance, significant voltage reduction ratios, low current ripple, and cost-effectiveness [4,5].

Non-isolated converters such as conventional buck circuits [6] exhibit structural simplicity and cost-effectiveness, yet their inherent limitations in high current ripple and restricted voltage step-down capability hinder deployment in high-voltage-differential, high-reliability applications. For improved circuit performance, Reference [7] introduces a tap-inductor buck converter that achieves reduced output current ripple in comparison with conventional topologies. However, this configuration necessitates isolated drivers, increasing implementation costs. Reference [8] employs a cascaded buck structure using dual switching modules to achieve higher voltage conversion ratios, though this approach elevates voltage stress on switching devices. Interleaved buck converters demonstrate fault-tolerant operation during switch failures, with Reference [9] implementing neural network-driven fault detection via nonlinear dimensionality reduction to maintain stable electrolyzer power delivery. Nevertheless, switching stress remains unresolved. Addressing this limitation, Reference [10] introduces a three-level interleaved buck architecture integrated with sliding mode control, effectively reducing device voltage stress while achieving enhanced dynamic response and system robustness.

Isolated converters overcome these constraints through the utilization of high-frequency transformers, providing both electrical isolation and significant step-down voltage ratios, thereby mitigating voltage stress on electrolyzers. Simultaneously, resonant topologies enable soft-switching operation, minimizing switching losses and enhancing conversion efficiency. Reference [11] demonstrates a half-bridge configuration in wind–PV–hydrogen storage systems, offering low switching loss and broad voltage regulation. Reference [12] further develops a half-bridge LLC resonant converter achieving full soft-switching with high efficiency, though this topology suffers from limited fault tolerance and elevated transformer current stress. To expand operational flexibility, Reference [13] implements a full-bridge LLC resonant converter with wide output voltage range and full-range zero-voltage switching (ZVS), improving hydrogen production efficiency while introducing significant resonant tank circulating currents. Reference [14] proposes a push–pull DC/DC converter featuring low current ripple and high conversion ratios, yet its complex center-tapped transformer design constrains applicability in high-power hydrogen production scenarios.

To address shortcomings in single-stage configurations, two-stage converter topologies have been specifically engineered for hydrogen production systems, offering enhanced operational capabilities through improved power conversion architectures. This architecture enhances system performance through functional specialization: the front stage enables wide-range voltage regulation, while the rear stage provides electrical isolation and ripple suppression. Reference [15] introduces a DCX two-stage DC/DC converter demonstrating high voltage conversion ratios and efficiency while requiring constant-current source operation and exhibiting elevated current ripple that necessitates additional filtering. Building on this foundation, Reference [16] incorporates a partial-power pre-regulator to achieve extended voltage input ranges and enhanced step-down capabilities. Reference [17] develops a two-stage Boost LLC series resonant converter that eliminates duty cycle loss in the secondary stage, improves efficiency, and facilitates direct electrolyzer integration with renewable energy system DC buses. Nevertheless, the control complexity inherent in dual-stage operation remains a critical challenge.

Controller design plays a critical role in hydrogen production converters. References [18,19] employ dual-loop PI control for electrolyzer voltage/current regulation. However, this approach depends on integral action to eliminate steady-state errors, resulting in delayed response to abrupt load disturbances. Moreover, sensitivity to parameter variations and nonlinearities compromises control robustness. To address these limitations, this work proposes a novel hybrid control strategy integrating Linear Active Disturbance Rejection Control (LADRC) with PI architecture. The outer LADRC voltage loop enhances disturbance rejection through active compensation, while the inner loop implements a synchronous current-balancing algorithm. This configuration significantly improves dynamic response during abrupt load transitions.

This paper is based on a cascaded power conversion architecture combining a two-phase interleaved buck converter with a three-phase interleaved LLC resonant converter. The front-stage interleaved buck topology achieves wide input voltage range operation, while the rear-stage multi-phase interleaved LLC configuration effectively minimizes output current ripple. By leveraging the DC transformer (DCX) mode of the LLC stage, control complexity is substantially reduced. A hybrid LADRC-PI control architecture is implemented, where the outer LADRC loop ensures strong disturbance rejection and the inner PI loop maintains current balance, achieving high conversion efficiency and low ripple output across full load conditions. The proposed system delivers a robust solution for hydrogen production power systems, featuring wide input voltage adaptability and enhanced operational reliability.

The principal contributions of this work are summarized as follows:

1. Development of a hybrid LADRC-PI control strategy with dual-loop compensation;
2. Co-optimization of power stage configuration and control algorithms to enable wide-range input voltage operation;
3. Experimental validation of the converter’s operational performance under full-load conditions.

## 2. Topology and Working Principle

**Figure 1:**
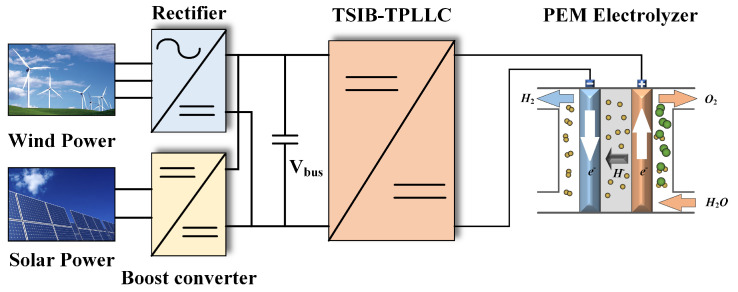
System configuration diagram.

The two-stage hybrid DC/DC converter (TSIB-TPLLC) is implemented in a renewable energy-integrated hydrogen generation system combining wind and photovoltaic power sources. As depicted in Figure 1, the system architecture comprises wind turbine generators with active rectifiers, photovoltaic arrays employing MPPT-enabled boost converters, the TSIB-TPLLC converter, and a proton exchange membrane (PEM) electrolyzer.

**Figure 2:**
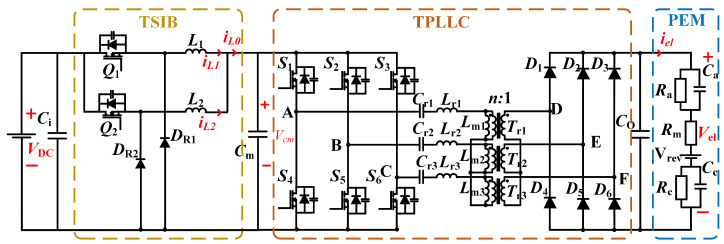
System architecture of the proposed two-stage converter.

The two-stage converter architecture, depicted in Figure 2, employs a front-stage two-phase interleaved buck converter with a centrally positioned regulation capacitor Ci. The subsequent stage integrates a three-phase interleaved LLC resonant converter operating in DC transformer (DCX) mode, functioning as an isolated voltage adaptation unit. The output stage interfaces with a low-voltage, high-current equivalent circuit model representing proton exchange membrane (PEM) electrolyzers.

### 2.1. PEM Electrolyzer Equivalent Circuit

The hydrogen production efficiency and dynamic response of proton exchange membrane (PEM) electrolyzers are critically dependent on their electrical characteristics. Accurate modeling of these systems requires an equivalent circuit representation that captures both steady-state behavior and transient dynamics. Such comprehensive modeling is essential for proper converter design and control strategy development [20,21,22].

**Figure 3:**
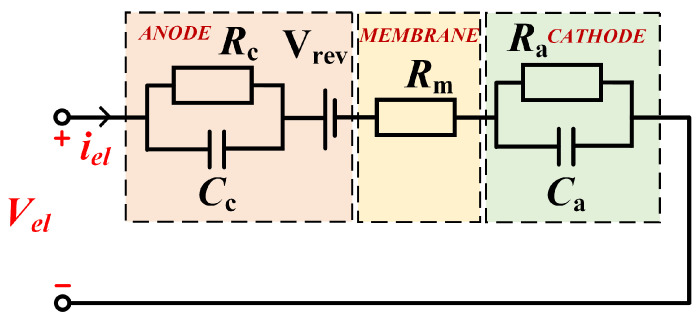
Equivalent circuit model of PEM electrolyzer.

The equivalent circuit model for a typical proton exchange membrane (PEM) electrolyzer, as illustrated in Figure 3, integrates three primary components: a reversible voltage source $V_{rev}$ representing thermodynamic activation potential, a membrane resistance $R_{m}$ modeling ohmic losses, and electrode-specific dynamic response units ($R_{a}$-$C_{a}$ and $R_{c}$-$C_{c}$) characterizing cathode/anode polarization effects. The reversible voltage E, governed by temperature, pressure, and catalyst activity, quantifies the minimum energy required to initiate water electrolysis. The membrane resistance $R_{m}$, which exhibits an exponential decrease with rising temperature, accounts for proton transport losses across the electrolyte. Detailed parameter values for this equivalent circuit are provided in Table 1.

The electrolyzer produces hydrogen by volume:

(1) $$V_{H_{2}}=\frac{\eta_{F}\cdot n_{e}\cdot I}{2F}$$

where $V_{H_{2}}$ is the hydrogen volumetric flow rate (m^3^/s), $\eta_{F}$ is the Faraday efficiency, $n_{e}$ is the number of single electrolytic cells in series, and *I* is the input current.

The efficiency of the PEM electrolyzer is as follows:

(2) $$\eta_{PEM}=\frac{V_{rev}\;I_{el}}{\left(V_{rev}I_{el}\right)+\left(R_{c}+R_{m}+R_{a}+\right){I_{el}}^{2}}$$

The resistance $R_{m}$ indicates the losses present in the electrolyzer membrane, and $R_{c}$ and $R_{a}$ indicate the behavior in the cathode and anode.

### 2.2. Front-Stage Two-Phase Interleaved Buck Circuit

**Figure 4:**
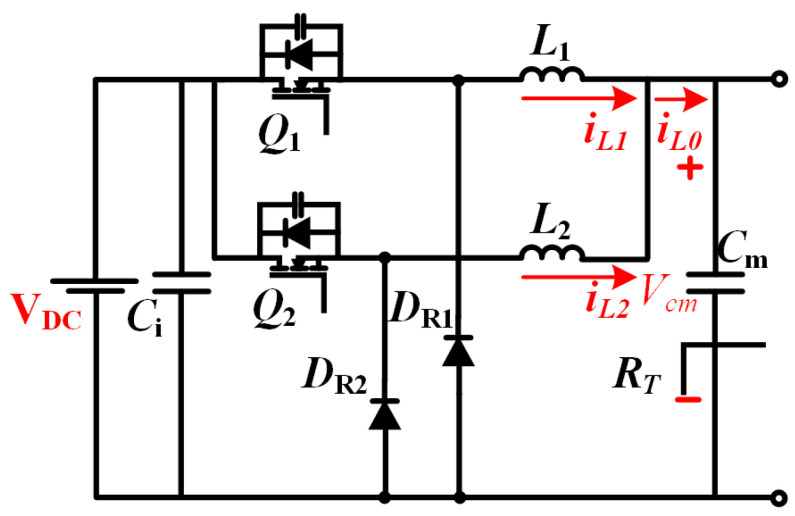
Two-phase interleaved buck circuit.

The front-stage circuit configuration, shown in Figure 4, employs parallel-connected classical single-phase buck converters. The topology features input voltage $V_{DC}$, input filter capacitor $C_{i}$, intermediate bus voltage $V_{m}$, and intermediate filter capacitor $C_{m}$. The load network comprises the total equivalent resistance $R_{T}$ representing the subsequent-stage converter and PEM electrolyzer stack. Key components include power switches $Q_{1}$ and $Q_{2}$, freewheeling diodes DR1–DR2, and interleaved inductors $L_{1}$ and $L_{2}$ with respective phase currents $i_{L1}$ and $i_{L2}$. The combined output current $i_{L}$ delivers power to the electrolyzer load [23,24].

**Figure 5:**
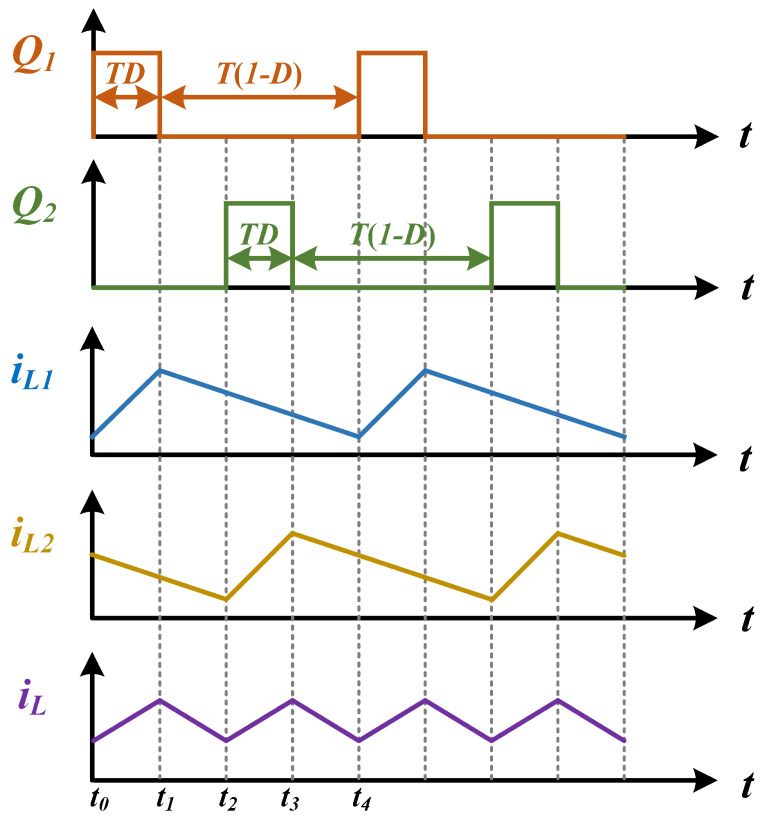
Ideal timing diagram of interleaved BUCK.

This analytical approach is predicated on ideal-type operational assumptions, thereby omitting consideration of parasitic effects inherent in circuit components and interconnect structures, along with variations in ambient operating conditions. As illustrated in Figure 5, the dual-switch configuration ($Q_{1}$ and $Q_{2}$) operates with identical duty cycles while maintaining strict 180° phase-synchronized interleaving. This symmetrical drive signal implementation ensures balanced current sharing between the parallel converter legs through complementary switching actions [25].

**Figure 6:**
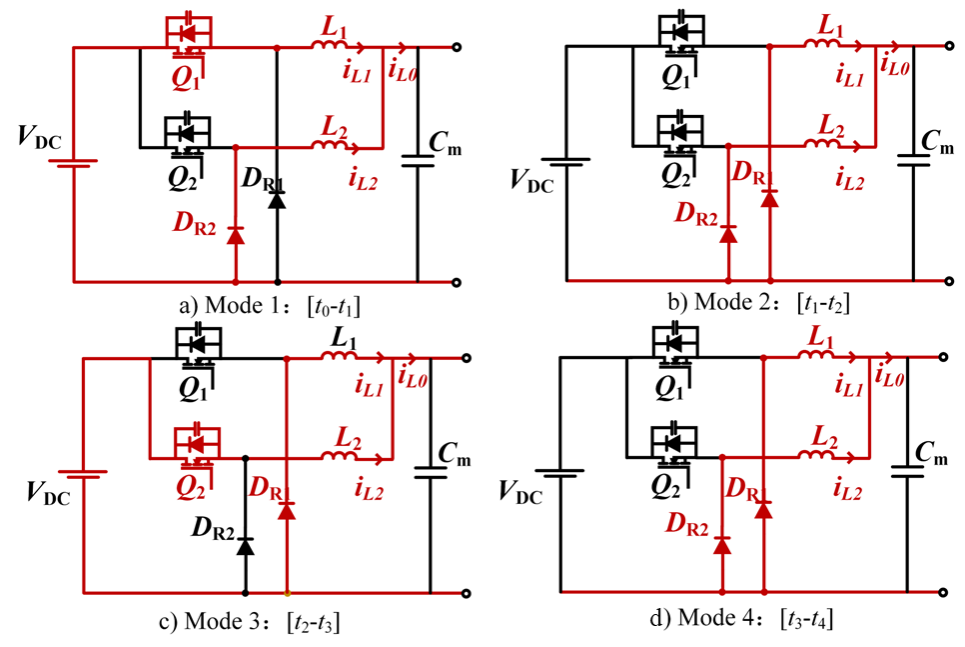
Equivalent circuit for each mode of interleaved BUCK.

The interleaved phase configuration achieves equal current sharing between parallel branches, reducing individual switch current stress and lowering device current ratings. Through interleaved phase cancellation, the output current ripple amplitude is effectively minimized while doubling the resultant ripple frequency compared to single-phase operation. This operational characteristic enables simplified output filter design and enhanced system dynamic response [26]. Operating in continuous conduction mode (CCM), the converter transitions through four distinct operational modes per switching cycle, as detailed in Figure 6.

(a) Mode 1: $Q_{1}$ turns on, $D_{2}$ renews current, $Q_{2}$ and $D_{R1}$ cut off, *L* stores energy while current rises, and $C_{o}$ charges.
(b) Mode 2: $Q_{1}$, $D_{2}$ are cut off, $D_{R1}$, $D_{R2}$ continue current, $L_{1}$ and $L_{2}$ current decreases, $C_{o}$ discharges.
(c) Mode 3: $Q_{2}$ turns on, $D_{R1}$ continues, $Q_{1}$, $D_{R2}$ cut off, *L* stores energy while current rises, $C_{o}$ charges.
(d) Mode 4: $Q_{1}$, $Q_{2}$ are cut off, $D_{R1}$, $D_{R2}$ renew current, current of $L_{1}$ and $L_{2}$ decreases, $C_{o}$ discharges.

The two-phase inductor currents $i_{L1}$, $i_{L2}$ and the intermediate capacitor voltage $V_{cm}$ are selected as state variables in conjunction with the operating modes of the circuit:

(3) $$x=\left[\begin{array}{c} i_{L1}\\ i_{L2}\\ v_{cm} \end{array}\right]$$

Based on Figure 6 and the relationship between inductor voltage and capacitor current, the state-space model is organized in the following equation:

(4) $$\left\{\begin{array}{cc} \frac{di_{L1}}{dt}=\frac{DV_{DC}-V_{Cm}}{L_{1}} & \\ \frac{di_{L2}}{dt}=\frac{DV_{DC}-V_{Cm}}{L_{2}} & \\ \frac{dv_{cm}}{dt}=\frac{i_{L1}+i_{L2}}{C_{m}}-\frac{v_{cm}}{C_{m}R_{T}} & \end{array}\right.$$

To analyze the perturbation response of the system, the state equations are linearized for small signals:

(5) $$\left\{\begin{array}{c} D=D_{0}+\tilde{d}\\ V_{DC}=V_{DC0}+{\tilde{V}}_{DC}\\ i_{L1}=I_{L10}+{\tilde{i}}_{L1}\\ i_{L2}=I_{L20}+{\tilde{i}}_{L2}\\ v_{cm}=V_{cm0}+{\tilde{V}}_{cm} \end{array}\right.$$

where the subscript “0” denotes the steady-state value and the wave symbol “∼” denotes the small signal perturbation component. When the dynamic term is ignored, the steady state equation is

(6) $$\left\{\begin{array}{c} D_{0}V_{DC0}=V_{cm0}\\ I_{L10}+I_{L20}=\frac{V_{cm0}}{R_{T}} \end{array}\right.$$

The small-signal model is obtained by substituting the perturbation variables into the state equation and ignoring higher-order small terms:

(7) $$\left\{\begin{array}{cc} \frac{di_{t1}}{dt}=\frac{D_{0}v_{DC}+V_{DC0}\tilde{d}-v_{cm}}{L_{1}} & \\ \frac{di_{t2}}{dt}=\frac{D_{0}v_{DC}+V_{DC0}\tilde{d}-v_{cm}}{L_{2}} & \\ \frac{d{\tilde{v}}_{cm}}{dt}=\frac{{\tilde{v}}_{L1}+{\tilde{v}}_{L2}}{C_{m}}-\frac{{\tilde{v}}_{cm}}{C_{m}R_{T}} & \end{array}\right.$$

We represent the small-signal model as a matrix form:

(8) $$\left\{\begin{array}{cc} \dot{x}=Ax+Bu\\ \; & y=Cx \end{array}\right.$$

There are

(9) $$\dot{x}=\frac{d}{dt}\left[\begin{array}{c} {\tilde{i}}_{L1}\\ {\tilde{i}}_{L2}\\ {\tilde{v}}_{cm} \end{array}\right],A=\left[\begin{array}{ccc} 0 & 0 & -\frac{1}{L_{1}}\\ 0 & 0 & -\frac{1}{L_{2}}\\ \frac{1}{C_{m}} & \frac{1}{C_{m}} & -\frac{1}{C_{m}R_{T}} \end{array}\right],B=\left[\begin{array}{cc} \frac{D_{0}}{L_{1}} & \frac{V_{0co}}{L_{1}}\\ \frac{D_{0}}{L_{2}} & \frac{V_{0co}}{L_{2}}\\ 0 & 0 \end{array}\right],u=\left[\begin{array}{c} {\tilde{v}}_{DC}\\ \tilde{d} \end{array}\right],C=\left[\begin{array}{ccc} 0 & 0 & 1 \end{array}\right]$$

### 2.3. Back-Stage Three-Phase Interleaved Parallel LLC-DCX Circuit

**Figure 7:**
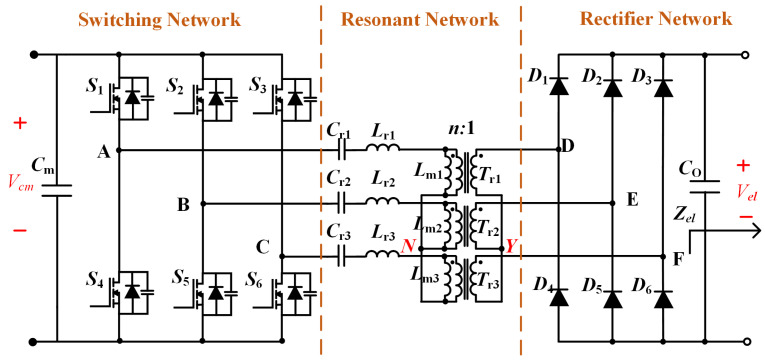
Three-phase interleaved LLC circuit.

The three-phase LLC resonant converter architecture, illustrated in Figure 7, employs three independent modules with star-connected (Y-connected) primary windings. Three half-bridge inverters are configured to produce 120° phase-shifted high-frequency square waves, employing interleaved gate driving to elevate the effective ripple frequency while minimizing amplitude. Resonant tank parameters ($L_{r}$, $C_{r}$) are optimized for DC transformer (DCX) mode operation, ensuring fixed voltage gain and high-efficiency power transfer. Secondary-side full-bridge rectification combined with three-phase interleaving triples the output current ripple frequency relative to single-module operation. This multi-phase synergistic design achieves high-frequency isolation, low conduction losses, and enhanced EMI immunity, fulfilling the low ripple, high-reliability requirements of electrolyzer applications [27].

**Figure 8:**
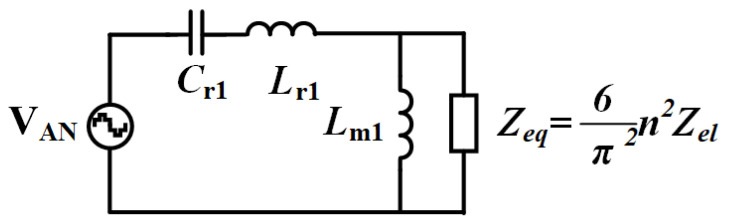
A-phase fundamental equivalent circuit.

The A-phase LLC circuit operation was analyzed using the fundamental harmonic approximation method under idealized conditions, excluding switching dead-time effects and parasitic components. The analysis assumes a quality factor sufficiently high to suppress higher-order harmonics, retaining only the fundamental frequency components. This first-order approximation yields the simplified equivalent circuit shown in Figure 8, where the resonant tank behavior is modeled using equivalent AC circuit theory with normalized voltage conversion characteristics.

The equivalent load $Z_{el}$ of the electrolyzer is folded to the original side by fundamental wave analysis:

(10) $$\begin{array}{cc} \; & Z_{eq}=\frac{6}{\pi^{2}}n^{2}Z_{el}\\ \; & =\frac{6}{\pi^{2}}n^{2}\left(\frac{V_{rev}}{I_{el}}+\frac{R_{a}}{j\omega R_{a}C_{a}+1}+R_{m}+\frac{R_{c}}{j\omega R_{c}C_{c}+1}\right) \end{array}$$

According to the single-phase fundamental equivalent circuit, the transfer function from the input to the output of the converter is obtained as

(11) $$\begin{array}{cc} \; & M\left(j\omega_{s}\right)=\frac{nV_{el}\left(j\omega_{s}\right)}{V_{cm}\left(j\omega_{s}\right)}=\frac{nV_{\mathrm{DY}}\left(j\omega_{s}\right)}{V_{\mathrm{AN}}\left(j\omega_{s}\right)}\\ \; & =\frac{Z_{eq}//j\omega_{s}L_{\mathrm{ml}}}{\frac{1}{j\omega_{s}C_{\mathrm{rl}}}+j\omega_{s}L_{\mathrm{rl}}+Z_{eq}//j\omega_{s}L_{\mathrm{ml}}} \end{array}$$

where $V_{DY}$ is the voltage from point D on the secondary side of the transformer to the star-wired neutral point Y in Figure 7.

Normalization is performed and modulo can be obtained:

(12) $$M\left(f_{n},\gamma ,K\right)=\frac{1}{\sqrt{{\left[\left(1-\frac{1}{f_{n}^{2}}\right)\gamma f_{n}\right]}^{2}+{\left[\left(1-\frac{1}{f_{n}^{2}}\right)\frac{1}{K}+1\right]}^{2}}}$$

The expressions for the normalized frequency $f_{n}$, the quality factor γ, and the inductance coefficient *K* are

(13) $$\left\{\begin{array}{c} f_{n}=\frac{f_{s}}{f_{\mathrm{rl}}}=\frac{\omega_{s}}{2\pi f_{\mathrm{rl}}}\\ \gamma =\frac{Z_{0}}{Z_{\mathrm{eq}}}\\ K=\frac{L_{\mathrm{ml}}}{L_{\mathrm{rl}}}\\ f_{\mathrm{rl}}=\frac{1}{2\pi \sqrt{L_{\mathrm{rl}}C_{\mathrm{rl}}}}\\ Z_{0}=\sqrt{\frac{L_{\mathrm{rl}}}{C_{\mathrm{rl}}}} \end{array}\right.$$

**Figure 9:**
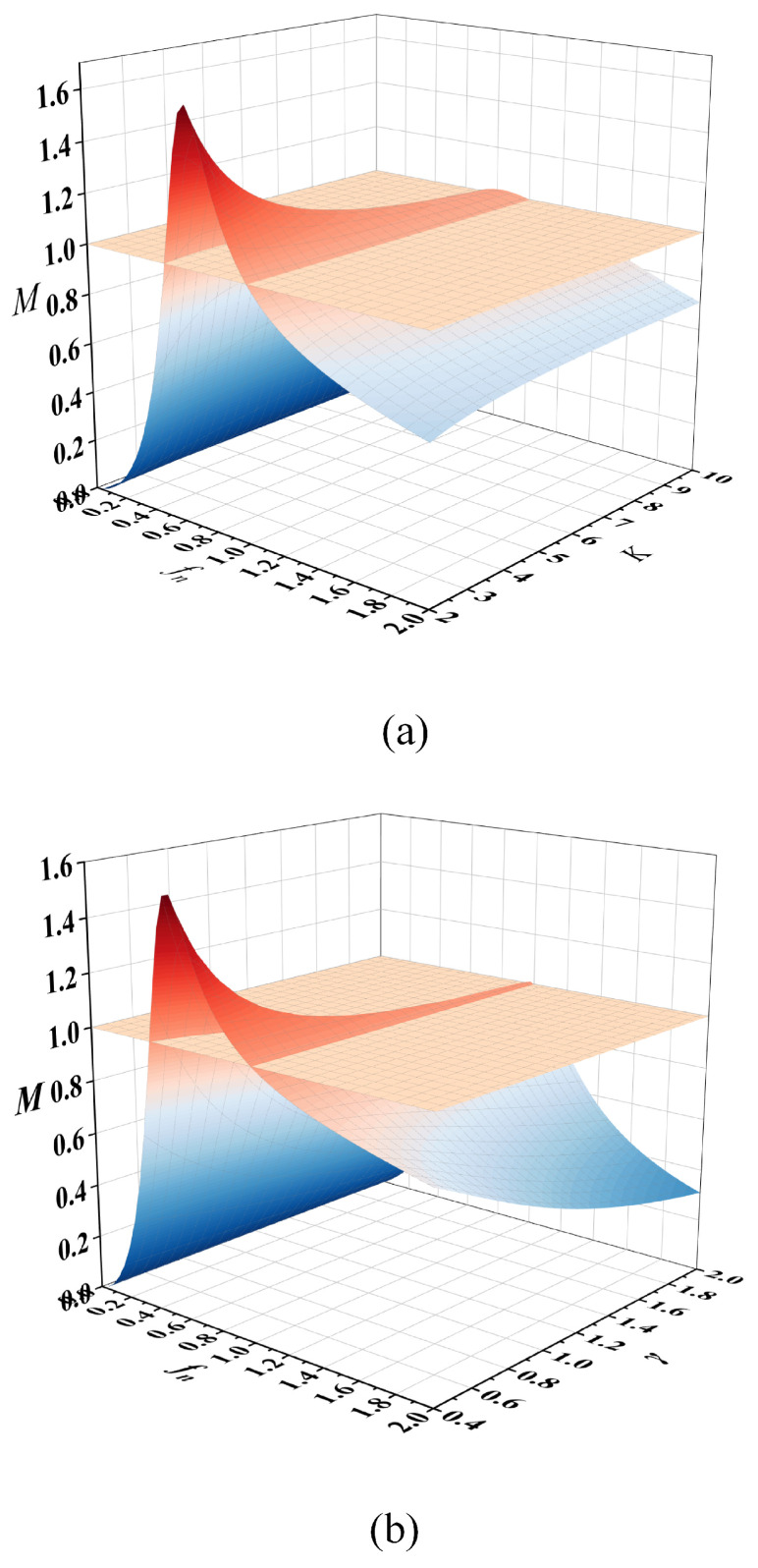
Gain surface of LLC converter: (**a**) fixed *K* gain surface; (**b**) fixed γ gain surface.

Figure 9 presents the three-dimensional gain characteristics of the three-phase LLC resonant converter under parameter variations. As shown in Figure 9a, the voltage gain surfaces corresponding to different inductance ratios (*K*) are analyzed with fixed γ = 0.6. Notably, all gain curves intersect precisely at the normalized resonant frequency (fn = 1) with unity voltage gain (*M* = 1), demonstrating K-independent operation at this critical point. Similarly, Figure 9b examines the gain surfaces for various γ values while maintaining *K* = 4, revealing analogous intersection characteristics at (fn = 1, *M* = 1).

This consistent intersection phenomenon confirms that the converter’s voltage gain remains invariant to both *K* and γ variations when operating precisely at the normalized resonant frequency (fn = 1). The invariant operational point (fn = 1, *M* = 1) is therefore identified as the optimal inductive working point for the DC transformer (DCX) mode implementation in the three-phase LLC resonant converter design. This characteristic establishes fn = 1 as the preferred design operating point for back-stage converter applications requiring precise voltage regulation through resonant frequency operation.

**Figure 10:**
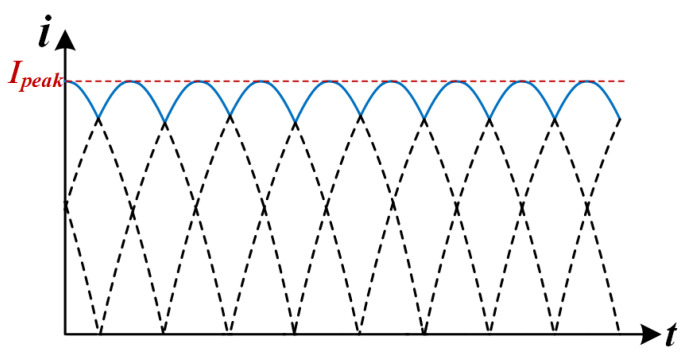
Three-phase staggered parallel LLC output current waveform before capacitor.

The output current ripple characteristics of the back-stage converter are analyzed by fundamental wave analysis [28]. Assuming that the converter operates in an ideal state (DCX mode), the waveform of the current before the output capacitor is shown in Figure 10.

The single-phase output current waveform can be approximated as an absolute value sinusoidal function:

(14) $$I_{SP}(t)=I_{\mathrm{peak}}\cdot \left|\sin(\omega t)\right|$$

where ω = 2π*f*, and $I_{peak}$ is the peak current. According to the law of conservation of energy, the output average current Io and the peak current satisfy the following relationship:

(15) $$I_{o}=\frac{1}{T}\int_{0}^{T}I_{SP}\left(t\right)dt=-\frac{6I_{\mathrm{peak}}}{T\omega }\cdot \cos\omega t{|}_{0}^{T/6}=\frac{3}{\pi }I_{\mathrm{peak}}$$

The peak current is solved for

(16) $$I_{\mathrm{peak}}=\frac{\pi }{3}I_{0}$$

After superposition of the three-phase currents, the instantaneous value of the total output current is

(17) $$I_{Total}\left(t\right)=\sum_{k=0}^{2}I_{SP}\left(t-k\cdot \frac{T}{3}\right)$$

The ripple frequency is boosted to 3$f_{s}$ due to the phase-interleaving effect, and the amplitude is significantly reduced due to waveform canceling. The output capacitor current $I_{c(t)}$ is the difference between the total current and the average current:

(18) $$I_{c}\left(t\right)=I_{Total}\left(t\right)-I_{o}$$

The RMS value of the ripple current is calculated by integrating the definition:

(19) $$I_{\mathrm{crms}}^{2}=\frac{1}{T}\int_{0}^{T}{\left[I_{Total}\left(t\right)-I_{o}\right]}^{2}dt$$

Substituting into the three-phase current expression and simplifying, the final RMS value of the ripple current is obtained:

(20) $$I_{\mathrm{crms}}\approx 0.045I_{0}$$

Whereas theoretical frameworks are predicated on ideal symmetry postulates, practical implementations integrate symmetric magnetic coupling topologies to compensate for inter-phase current deviations by enforcing balanced inductance parameters in multi-phase architectures. Additional ripple attenuation mechanisms include inherent damping from parasitic circuit elements, real-time perturbation compensation via dynamic control strategies, and resonant impedance adaptation induced by minor operational frequency deviations. Experimental validation confirms that these combined effects sufficiently suppress output current ripple amplitude, meeting the stringent low ripple requirements critical for hydrogen production in electrolyzer applications.

## 3. Proposed Control Strategy

**Figure 11:**
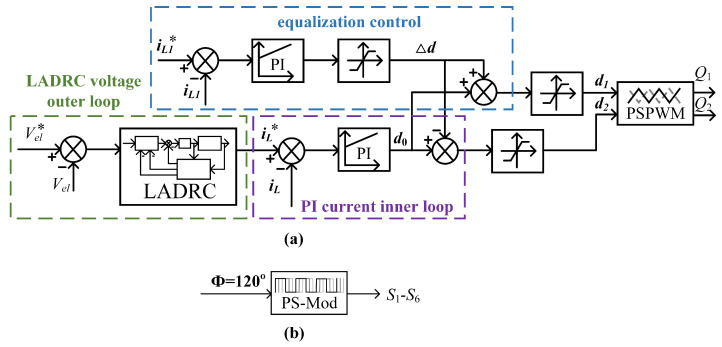
Overall block diagram of the proposed control strategy: (**a**) front-stage control strategy; (**b**) back-stage control strategy.

To address the stringent current ripple suppression and dynamic response requirements of proton exchange membrane (PEM) electrolyzers, this work proposes a three-tier control architecture integrating linear active disturbance rejection control (LADRC), PI regulation, and phase current balancing, as illustrated in Figure 11. The outer voltage loop employs LADRC for real-time total disturbance estimation and compensation, while the inner PI current loop ensures rapid reference tracking. This hierarchical structure achieves optimal balance between transient response and system robustness. The implemented phase current balancing methodology improves converter operational reliability through precise current-sharing maintenance in two-phase interleaved buck converter topologies, consequently enhancing power conversion efficiency during load transient performance.

### 3.1. LADRC Voltage Outer Loop

The linear active disturbance rejection control (LADRC) framework comprises three fundamental components: a linear tracking differentiator (LTD), a linear extended state observer (LESO), and a linear state error feedback (LSEF) controller [29,30]. For second-order LADRC implementation, the core architecture synthesizes the LESO with proportional-derivative (PD) control, enabling systematic parameterization through observer and controller bandwidth correlation. This bandwidth-coordinated design methodology effectively simplifies controller tuning while preserving disturbance rejection capabilities.

Equation (4) and $V_{cm}$ = *n*$V_{el}$ are obtained by association:

(21) $$\left\{\begin{array}{cc} \frac{di_{L1}}{dt}=\frac{DV_{DC}-nV_{el}}{L_{1}} & \\ \frac{di_{L2}}{dt}=\frac{DV_{DC}-nV_{el}}{L_{2}} & \\ \frac{dV_{el}}{dt}=\frac{i_{L1}+i_{L2}}{nC_{m}}-\frac{V_{el}}{C_{m}R_{T}} & \end{array}\right.$$

where there is $i_{L}$ = $i_{L1}$ + $i_{L2}$, a derivative for ${\dot{V}}_{el}$:

(22) $$\begin{array}{cc} \; & {\ddot{V}}_{el}=\frac{d}{dt}\left(\frac{i_{L}}{nC_{m}}-\frac{V_{el}}{C_{m}R_{T}}\right)\\ \; & =\frac{1}{nC_{m}}\frac{di_{L}}{dt}-\frac{1}{C_{m}R_{T}}{\dot{V}}_{el} \end{array}$$

Assuming that the voltage outer-loop control output is $i_{L}^{*}$ and introducing a perturbation f yields

(23) $${\ddot{V}}_{ei}=b_{0}u+\left(\frac{1}{nC_{m}}\frac{di_{L}}{dt}-\frac{{\dot{V}}_{ei}}{C_{m}R_{T}}-\frac{1}{nC_{m}}u\right)$$

where $b_{0}=\frac{1}{nC_{m}}$, $u=i_{L}^{*}$, $f=\frac{1}{nC_{m}}\frac{di_{L}}{dt}-\frac{{\dot{V}}_{ei}}{C_{m}R_{T}}-\frac{1}{nC_{m}}u$.

We set the state variables as follows: $x_{1}=V_{el}$, $x_{2}={\dot{V}}_{el}$, $x_{3}=f$, $y=V_{el}$. The state-space expression for this system is

(24) $$\left\{\begin{array}{cc} \left[\begin{array}{c} {\dot{x}}_{1}\\ {\dot{x}}_{2}\\ {\dot{x}}_{3} \end{array}\right]=\left[\begin{array}{ccc} 0 & 1 & 0\\ 0 & 0 & 1\\ 0 & 0 & 0 \end{array}\right]\left[\begin{array}{c} x_{1}\\ x_{2}\\ x_{3} \end{array}\right]+\left[\begin{array}{cc} 0 & 0\\ b_{0} & 0\\ 0 & 1 \end{array}\right]\left[\begin{array}{c} u\\ \dot{f} \end{array}\right] & \\ y=x_{1} & \end{array}\right.$$

The corresponding third-order LESO is

(25) $$\left\{\begin{array}{cc} {\dot{z}}_{1}=z_{2}-\beta_{1}\left(z_{1}-V_{el}\right) & \\ {\dot{z}}_{2}=z_{3}-\beta_{2}\left(z_{1}-V_{el}\right)+b_{0}i_{L}^{*} & \\ {\dot{z}}_{3}=-\beta_{3}\left(z_{1}-V_{el}\right) & \end{array}\right.$$

where the observer gain $\beta_{1}=3\omega_{o},\beta_{2}=3\omega_{o}^{2},\beta_{3}=\omega_{o}^{3}$. The bandwidth $\omega_{0}$ determines the speed of disturbance estimation. $z_{1}$ tracks the electrolyzer voltage $V_{el}$ quickly, $z_{2}$ tracks ${\dot{V}}_{el}$, and $z_{3}$ tracks the total disturbance *f* of the system.

The reference value for the outer-loop output current $i_{L}^{*}$ is

(26) $$i_{L}^{*}=\frac{1}{b_{0}}\left[k_{P}\left(V_{el}^{*}-z_{1}\right)-k_{d}z_{2}-z_{3}\right]$$

**Figure 12:**
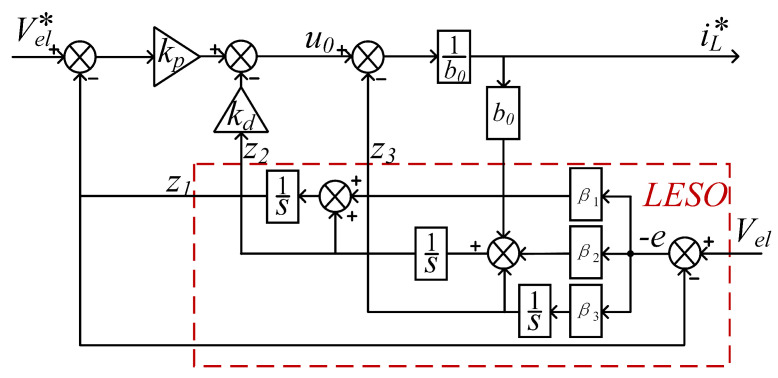
Block diagram of LADRC-based voltage outer-loop control.

Then the block diagram of the voltage outer-loop control system is shown in Figure 12.

When LSEF is controlled using PD, the pole configuration of the controller can be obtained by $k_{p}=\omega_{c}^{2},\;k_{d}=2\omega_{c}$. $\omega_{c}$ describes the controller bandwidth and is a key parameter in the LADRC control algorithm. It is used to characterize the dynamic response speed of the controller and its ability to suppress perturbations. The selection of a suitable gain $k_{p},k_{d}$ stabilizes the system.

### 3.2. PI Current Inner Loop and Equalization Loop

The inner current loop ensures rapid tracking of the reference current ($I_{ref}$) generated by the voltage outer loop, enabling swift adjustment of the output current to target values during input voltage fluctuations or abrupt load changes. In this study, the bandwidth is maintained at 1/10 of the switching frequency. Applying Laplace transform to the state equations in (3)–(9) yields the following transfer function:

(27) $$G_{id}\left(s\right)=\frac{{\tilde{i}}_{L}\left(s\right)}{\tilde{d}\left(s\right)}=\frac{2V_{DC0}}{LC_{m}s^{2}+\frac{L}{R_{T}}s+2}$$

We define the current error $e_{i}=i_{L}^{*}-i_{L}$, so the PI controller output duty cycle correction is

(28) $$d_{0}=K_{p}e_{i}+K_{i}\int e_{i}dt$$

where $K_{p}$ is the scale factor and $K_{i}$ is the integration factor.

**Figure 13:**
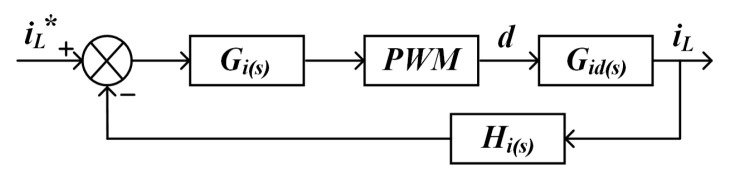
Block diagram of current inner-loop control.

The control block diagram of the current inner loop is shown in Figure 13.

The current loop open-loop transfer function can be obtained as

(29) $$T_{oi}\left(s\right)=G_{i}\left(s\right)G_{id}\left(s\right)PWM\left(s\right)$$

The closed-loop transfer function is

(30) $$T_{ci}\left(s\right)=\frac{G_{i}\left(s\right)G_{id}\left(s\right)PWM\left(s\right)}{1+G_{i}\left(s\right)G_{id}\left(s\right)PWM\left(s\right)H_{i}\left(s\right)}$$

$H_{i}\left(s\right)$ denotes the transfer function of the current feedback path, defined as the gain and dynamic characteristics of the sensor measurement link. The current sensor adopts a closed-loop Hall effect device, whose bandwidth is much higher than the system switching frequency, and the dynamic response can be approximated as the proportional link $H_{i}\left(s\right)=K_{sens}$. Meanwhile, the cutoff frequency of the first-order RC low-pass filter is much higher than the current loop bandwidth, and its phase delay can be neglected. Therefore the stability analysis of the closed-loop transfer function can be directly based on the unit feedback assumption ($H_{i}\left(s\right)=1$).

We define the equalization error $e_{s}=\Delta i=i_{L1}-i_{L2}$, so the equalization PI controller output duty cycle correction is

(31) $$\Delta d=K_{p-s}e_{s}+K_{i-s}\int e_{s}dt$$

where $K_{p-s}$, $K_{i-s}$ is the equalization control parameter.

### 3.3. Back-Stage LLC-DCX Control Strategy

The back-stage three-phase interleaved shunt LLC resonant converter is operated in DC transformer (DCX) mode to achieve fixed voltage gain and high-frequency electrical isolation. In DCX mode, the switching frequency fs is always synchronized with the resonant frequency $f_{r1}$.

(32) $$f_{s}=f_{r1}=\frac{1}{2\pi \sqrt{L_{r1}C_{r1}}}$$

It ensures that all MOSFETs achieve zero-voltage switching (ZVS), significantly reducing switching losses. This mode suppresses output voltage fluctuations through the dynamic characteristics of the resonant cavity. It utilizes a three-phase interleaved (120° phase difference) gate drive signal to achieve offsetting of harmonic ripple components.

## 4. Comparison and Analysis

Table 2 presents a comparative analysis between the two-stage topology adopted in this work and the single-stage topologies of existing studies. Through such a comparison, it is revealed that the proposed topology demonstrates greater suitability for hydrogen production scenarios powered by renewable energy. Specifically, it achieves a performance balance featuring “wide input regulation, low ripple output, fast dynamic response, and high-efficiency operation”, which provides critical technical support for efficient hydrogen production from intermittent renewable energy sources.

## 5. Experimental Results

**Figure 14:**
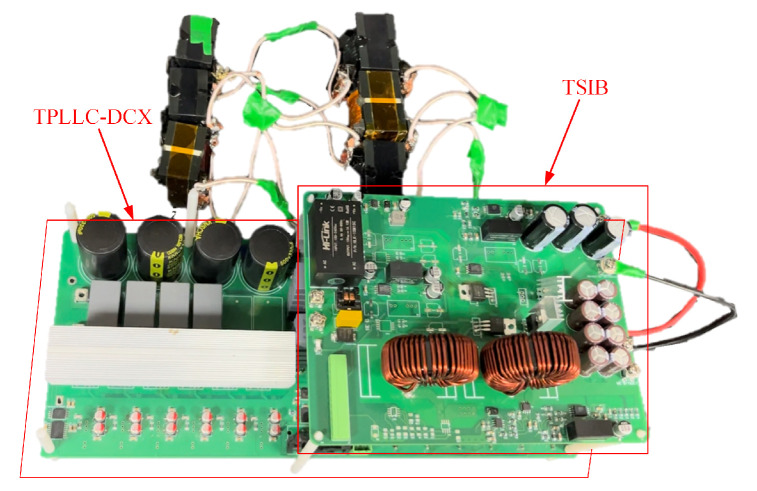
Experimental platform for the converter.

An experimental prototype rated at 1.2 kW was developed utilizing a DSP28335 microcontroller as the core control unit. The experimental platform is illustrated in Figure 14. The experimental parameters are shown in Table 3.

**Figure 15:**
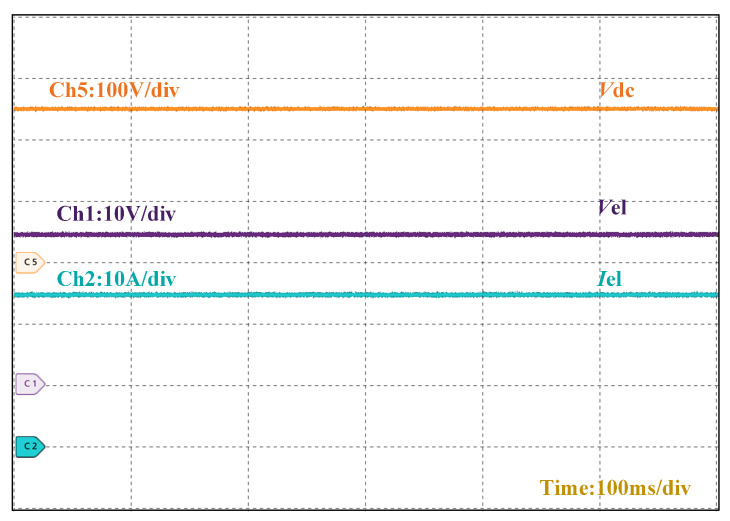
Output waveform of the converter at steady state.

Figure 15 demonstrates the steady-state operational waveforms of the converter. With a DC bus input voltage of 250 V, the output voltage stabilizes at 24 V while delivering 24.6 A to the electrolyzer, confirming the converter’s high step-down conversion capability.

**Figure 16:**
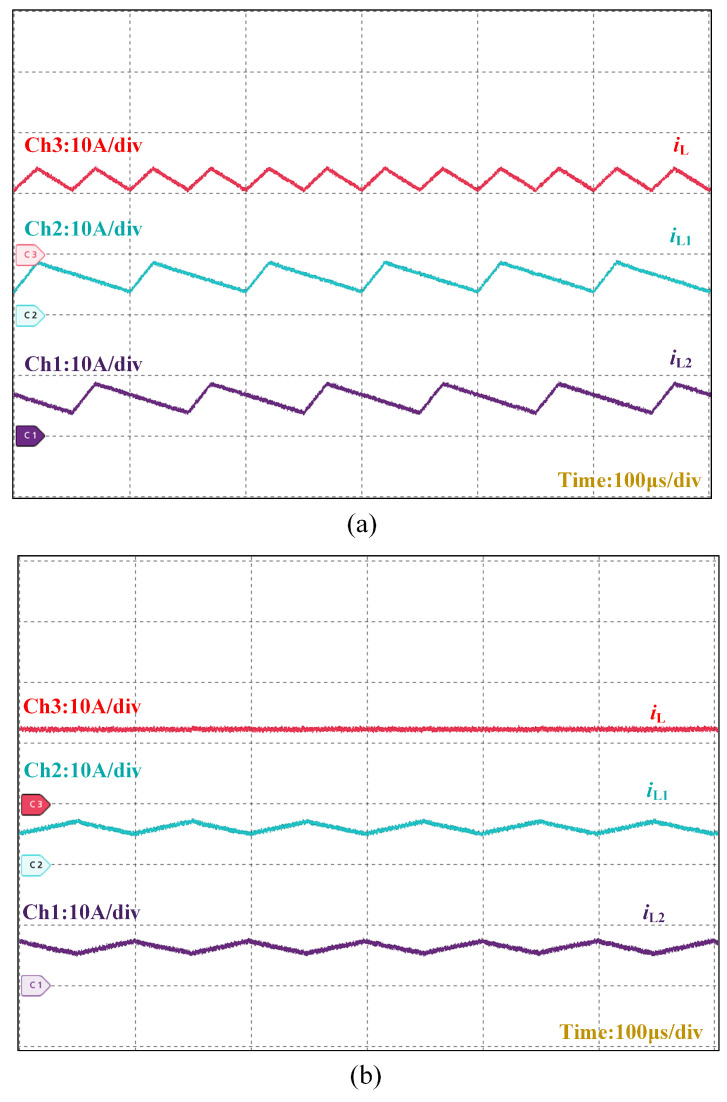
Experimental results of current in front-stage interleaved buck under different controls: (**a**) PI double closed loop control; (**b**) LADRC-PI control.

Figure 16 presents a comparative analysis of phase currents and total output current in the front-stage interleaved buck converter under different control strategies. The current per phase for the PI double closed-loop control is $\Delta i_{1}=i_{\max}-i_{\min}\approx 5.6$ A and for the LADRC-PI control method is $\Delta i_{2}=i_{\max}-i_{\min}\approx 2.2$ A. The LADRC-PI implementation demonstrates reduced phase current fluctuations and lower total output current ripple.

**Figure 17:**
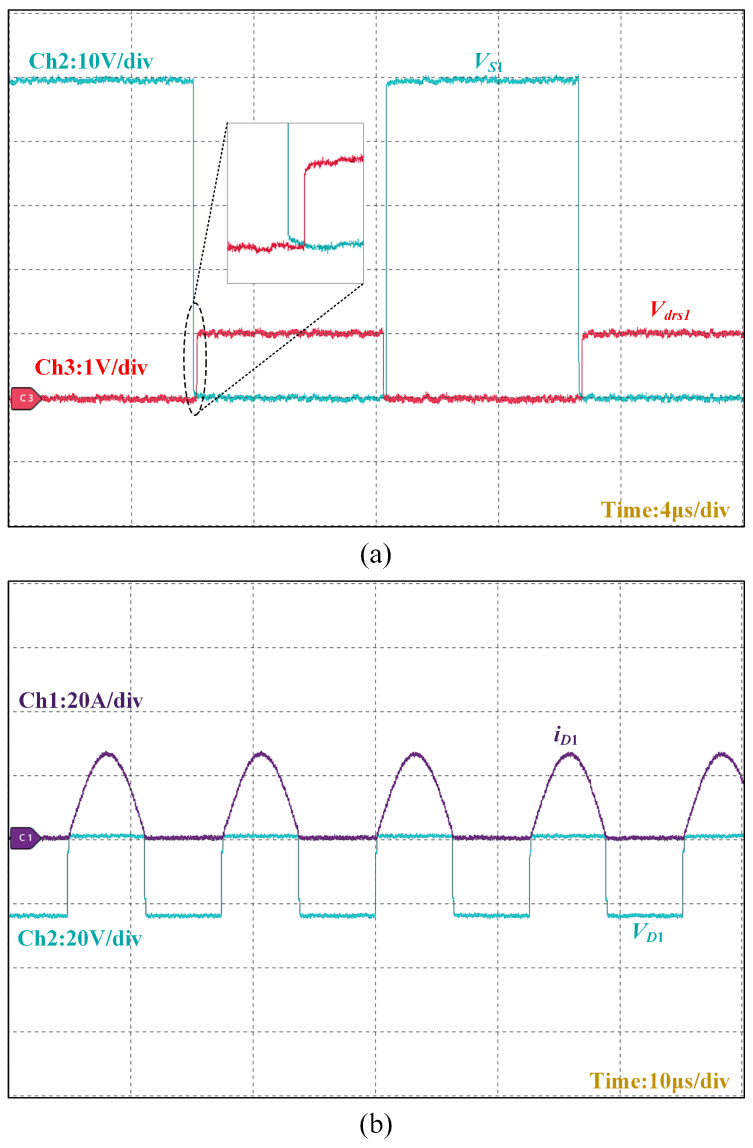
ZVS for $S_{1}$, ZCS for $D_{1}$: (**a**) $S_{1}$ switching tube voltage and drive signals; (**b**) $D_{1}$ switching tube voltage and current.

Figure 17a depicts that the voltage through the switching tube is zero when $S_{1}$ turns on before $S_{1}$ realizes ZVS. Figure 17b depicts that the current through the diode is zero when $D_{1}$ turns on before $D_{1}$ realizes ZCS.

**Figure 18:**
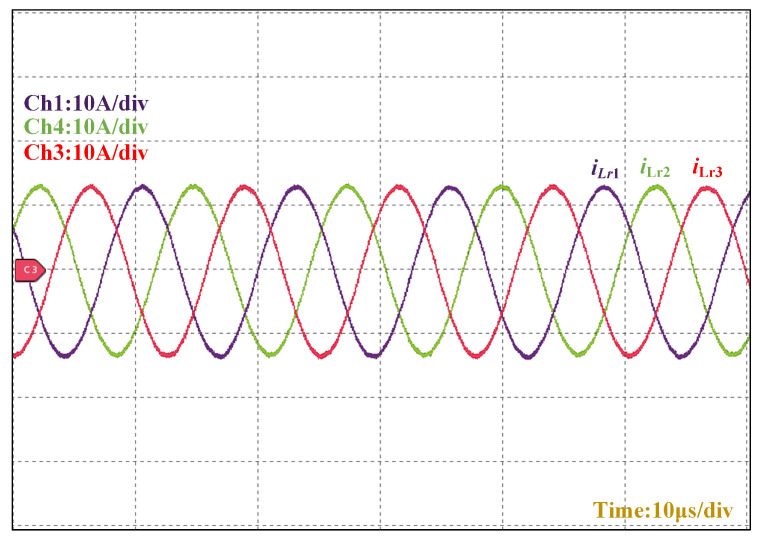
Three-phase LLC resonant inductor current.

Figure 18 illustrates the resonant inductor current ($i_{Lr}$) waveform in the three-phase interleaved LLC converter. The $i_{Lr}$ exhibits a sinusoidal profile when operating at the resonant frequency in DC transformer (DCX) mode, confirming optimal resonant tank operation through zero-phase-angle switching transitions. This characteristic sinusoidal current minimizes switching losses while maintaining voltage gain stability across the LLC stage.

**Figure 19:**
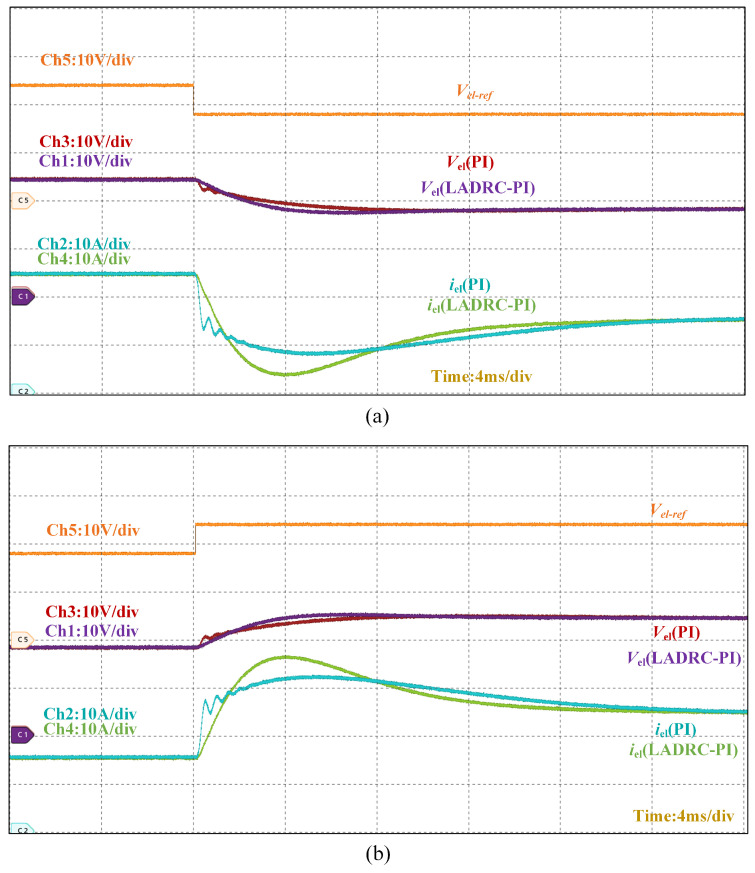
Output voltage–current waveform of the converter when the reference voltage changes. (**a**) Output voltage–current of the converter during reference voltage drop. (**b**) Output voltage–current of the converter when the reference voltage rises.

Figure 19 demonstrates the dynamic performance of the converter under reference voltage variations. The experimental results reveal a 12 ms response time for the PI-based dual-loop control compared to 8 ms for the LADRC-PI strategy, confirming the latter’s superior transient performance. When operating in steady state, the LADRC-PI implementation achieves reduced output current ripple amplitude while maintaining equivalent current tracking accuracy, as evidenced by the stabilized electrolyzer current waveform.

**Figure 20:**
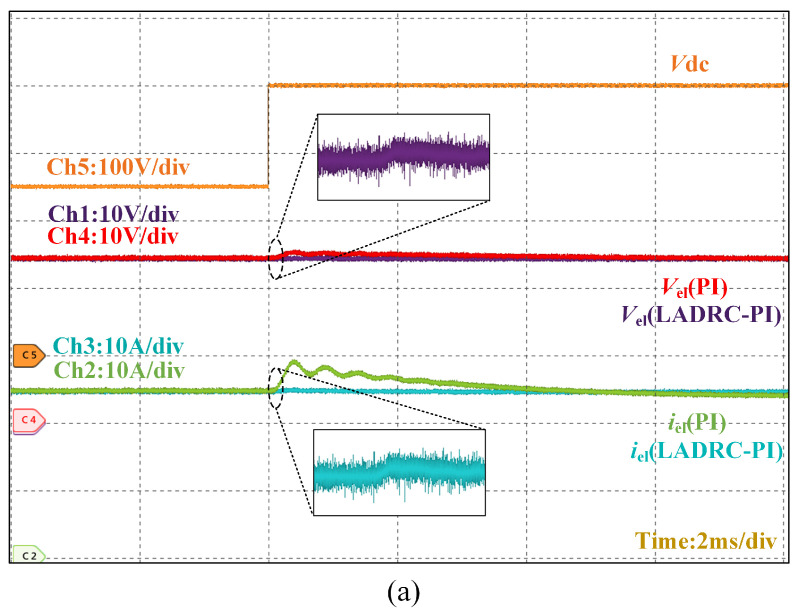

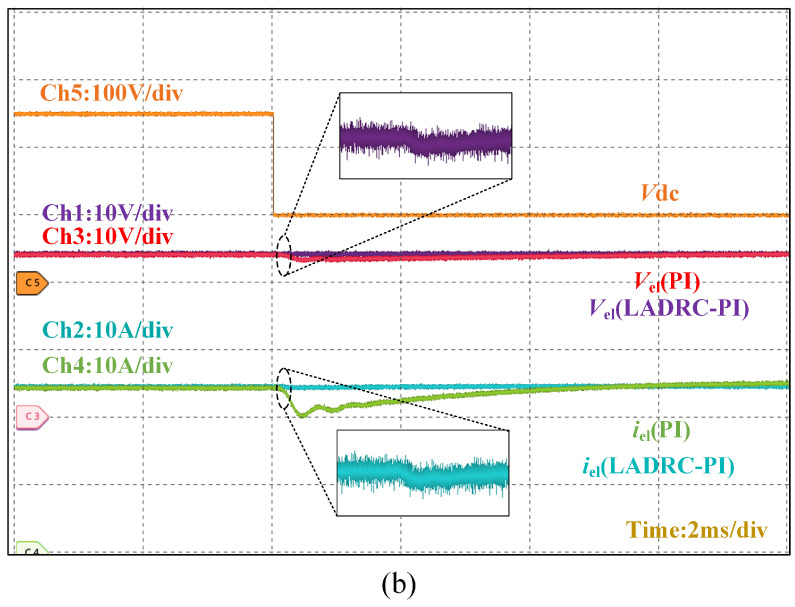
Converter output voltage–current waveforms during input voltage abrupt changes. (**a**) Sudden change in input voltage to 400 V. (**b**) Input voltage changes abruptly to 100 V.

Figure 20 describes how the output voltage–current of the converter is virtually unaffected when the output dc bus voltage fluctuates up and down.

**Figure 21:**
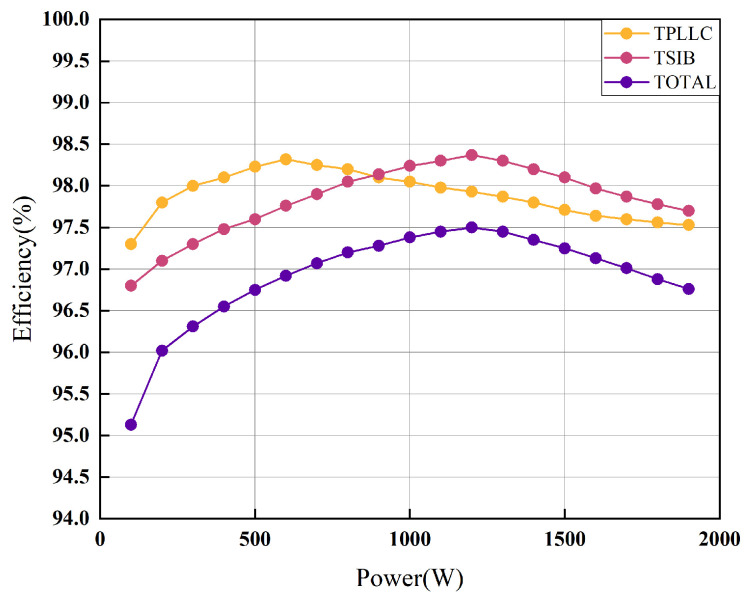
Overall efficiency of the secondary converter and the efficiency of each stage of the converter.

Figure 21 presents the efficiency characteristics of the secondary converter based on experimental results. The system achieves maximum efficiency of 97.5% under rated power conditions, demonstrating the converter’s capability to meet the stringent efficiency requirements for hydrogen production applications. This performance aligns with industrial demands for high-efficiency power conversion in electrolyzer systems.

## 6. Discussion

The TSIB-TPLLC converter with hybrid LADRC-PI control demonstrates significant advancements in addressing the critical requirements of PEM electrolyzer systems, including wide input voltage adaptability, ultra-low output current ripple, and enhanced dynamic performance. Experimental validation confirms that the LADRC-PI control architecture achieves a 33.3% improvement in transient recovery time (8 ms vs. 12 ms) compared to conventional dual-loop PI control during reference voltage steps. This improvement is attributed to the LADRC’s ability to actively estimate and compensate for system disturbances, thereby overcoming the sluggish response inherent in integral-based PI controllers. Additionally, the multi-phase interleaved topology reduces output current ripple to 0.5% through harmonic cancellation, a critical feature for prolonging PEM electrolyzer lifetime and improving hydrogen production efficiency.

The DCX mode operation of the three-phase LLC stage ensures fixed voltage gain and soft-switching transitions (ZVS/ZCS), aligning with resonant converter studies while surpassing them in fault tolerance and ripple suppression due to the interleaved design.

The co-design of topology and control strategies enables a balance between wide input voltage regulation (250–400 V) and ultra-low ripple output, advancing hydrogen power systems. While the hybrid control reduces phase current imbalance by 60% compared to conventional PI methods, further optimization of observer bandwidth and resonant tank parameters could enhance performance across extended voltage ranges. Scaling this architecture for multi-electrolyzer parallel operation necessitates decentralized control strategies and dynamic load-sharing mechanisms. Integrating AI-driven parameter tuning, as suggested in prior studies, could further improve adaptability to intermittent renewable energy inputs, aligning with global decarbonization goals. These technological advancements are poised to catalyze the evolution of cyber–physical coupled electrolytic hydrogen synthesis architectures, exhibiting enhanced operational compliance with the dynamic requirements of next-generation renewable energy grids.

## 7. Conclusions

This paper is based on a two-stage converter topology combining a two-phase interleaved buck converter with a three-phase interleaved LLC (TSIB-TPLLC) and a hybrid LADRC-PI control strategy to meet the high-efficiency, low-ripple DC/DC conversion requirements of proton exchange membrane (PEM) electrolyzer systems. The front-stage two-phase interleaved buck circuit doubles the output current ripple frequency through phase-interleaved operation and reduces current ripple via wide input voltage regulation. The three-phase interleaved LLC at the backstage operates in DC transformer (DCX) mode, suppressing the output ripple factor below 0.5% through multi-phase parallel cancellation while achieving ZVS/ZCS soft-switching for efficiency improvement. Experimental results demonstrate a stabilized 24 V output from a 250 V input, validating the topology’s high step-down ratio and robustness.

The control strategy integrates a second-order linear active disturbance rejection control (LADRC) outer loop with a PI inner loop. The LADRC outer loop estimates and compensates for internal and external disturbances in real time through an extended state observer, addressing the traditional PI control’s slow dynamic response. The PI inner loop optimizes current tracking accuracy and reduces phase current imbalance, enhancing system efficiency. This hybrid control balances dynamic performance and stability.

Through the co-design of topology and control, the proposed solution achieves wide input adaptability, high efficiency, and ultra-low ripple output. Future work will explore multi-electrolyzer parallel control, AI-based adaptive parameter tuning, and magnetic/resonant component optimization to improve power density and voltage range, advancing intelligent and integrated hydrogen production systems.

## Figures and Tables

**Table 1:** Equivalent circuit parameters.

Parameter	Symbol	Value
Film loss model resistor	$R_{m}$	0.188 Ω
Reverse elimination voltage	$V_{rev}$	8 V
Cathode activation loss model resistance	$R_{a}$	0.408 Ω
Cathode capacitance	$C_{a}$	38 F
Anode activation loss model resistance	$R_{c}$	0.055 Ω
Anode capacitance	$C_{c}$	38 F

**Table 2:** Comparison between two-stage and single-stage topologies.

Criteria	[31]	[32]	[33]	This Paper
Topology	SIBC	R-LLC	T-Type LLC	TSIB -TPLLC
Control strategy	ISMC	PFM	Smooth trajectory control	LADRC-PI
Input voltage range	Fixed	Fixed	Limited	Wide
Output current ripple	Low	Medium	Medium	Ultra-low
Complexity/cost	Low	High	Moderate	Moderate
Dynamic response	Fast	Moderate	Fast	Fast
Voltage gain	High	Ultrawide	Gain extended	Wide
Fault tolerance	High	Low	Moderate	Moderate
Efficiency	High	High	Relatively high	High

**Table 3:** Equivalent circuit parameters.

Parameter	Symbol	Value
Bus input voltage	$V_{DC}$	250 V
Input capacitance	$C_{i}$	100 μF
Energy storage inductors	*L*	833 μH
BUCK switching frequency	$f_{s1}$	10 khz
Intermediate regulator capacitor	$C_{m}$	200 μF
Resonant inductors	$L_{r}$	18 μH
Resonant capacitor	$C_{r}$	220 nF
Magnetic inductance	$L_{m}$	72 μH
LLC switching frequency	$f_{s2}$	80 khz
Output capacitors	$C_{o}$	1600 μF

## Data Availability

The data presented in this study are available on request from the corresponding author due to privacy.

## Abbreviations

The following abbreviations are used in this paper:

TSIB-TPLLC	Two-stage interleaved buck three-phase LLC
LADRC-PI	Linear active disturbance rejection control-proportional-integral
PEM	Proton exchange membrane
DCX	DC transformer
ZVS	Zero voltage switching
ZCS	Zero current switching
CCM	Continuous conduction mode
LESO	Linear extended state observer
LSEF	Linear state error feedback
RMS	Root mean square
EMI	Electromagnetic interference
SIBC	Stacked interleaved buck
ISMC	Integral sliding mode control
PFM	Pulse frequency modulation
